# Imaging of surface spin textures on bulk crystals by scanning electron microscopy

**DOI:** 10.1038/srep37265

**Published:** 2016-11-22

**Authors:** Hiroshi Akamine, So Okumura, Sahar Farjami, Yasukazu Murakami, Minoru Nishida

**Affiliations:** 1 Department of Applied Science for Electronics and Materials, Interdisciplinary Graduate School of Engineering Sciences, Kyushu University, Kasuga 816-8580, Japan; 2 Department of Engineering Sciences for Electronics and Materials, Faculty of Engineering Sciences, Kyushu University, Kasuga 816-8580, Japan; 3 Department of Applied Quantum Physics and Nuclear Engineering, Faculty of Engineering, Kyushu University, Fukuoka 819-0395, Japan; 4 The Ultramicroscopy Research Center, Kyushu University, Fukuoka 819-0395, Japan

## Abstract

Direct observation of magnetic microstructures is vital for advancing spintronics and other technologies. Here we report a method for imaging surface domain structures on bulk samples by scanning electron microscopy (SEM). Complex magnetic domains, referred to as the maze state in CoPt/FePt alloys, were observed at a spatial resolution of less than 100 nm by using an in-lens annular detector. The method allows for imaging almost all the domain walls in the mazy structure, whereas the visualisation of the domain walls with the classical SEM method was limited. Our method provides a simple way to analyse surface domain structures in the bulk state that can be used in combination with SEM functions such as orientation or composition analysis. Thus, the method extends applications of SEM-based magnetic imaging, and is promising for resolving various problems at the forefront of fields including physics, magnetics, materials science, engineering, and chemistry.

Understanding magnetic domain structures, which are sometimes referred to as spin textures, is essential for both basic science and technological applications. In the past decade, significant advances have been made in magnetic imaging in areas of research such as magnetic recording media^1^,^2^ and permanent magnets^3^,^4^. Magnetic domains have been imaged by magnetic force microscopy (MFM)^5^,^6^, scanning electron microscopy polarisation analysis (SEMPA)^7^,^8^, magneto-optical Kerr microscopy^9^,^10^, and techniques using a transmission electron microscope (TEM) (*i.e.,* Lorentz microscopy and electron holography)^11^,^12^,^13^,^14^,^15^. However, despite being successfully applied to many systems, these technologies still have drawbacks (depending on the principles of experiments) such as a limited field of view, long measurement time, ultra-high vacuum conditions, and preparation of a thin-foil specimen. Although we need to understand the relationships between the magnetic domain structures and other crystal factors, such as chemical composition, crystal orientations, lattice strains, and morphology, the conventional methods, except for TEM observations, are not always convenient for these multidisciplinary studies. Further development of techniques for magnetic imaging is needed.

Here, we focus on using scanning electron microscopy (SEM), which does not employ any special probes such as spin-polarised electrons. Conventional SEM provides a simple method of magnetic imaging, referred to as type-I and type-II imaging^9^,^16^,^17^. The magnetic contrast originates from the deflection of secondary and/or backscattered electrons emitted from the sample. This deflection is the result of the Lorentz force from the stray magnetic field and/or inner magnetization of the sample, and modifies the collection efficiency of secondary/backscattered electrons. The image contrast thus represents the magnetic domains. However, the asymmetric geometry of the Everhart-Thornley detector (ETD), which has been used for conventional type-I and type-II imaging, can only visualise limited orientations of magnetic domains. To solve this key problem, we used SEM with a symmetric annular in-lens detector (ILD) (Fig. 1). As discussed below in greater detail, the ILD provides an easy way to visualise a surface domain structure over a wide region, even for complex magnetic domains such as the mazy patterns produced in uniaxial Co-Pt and Fe-Pt alloys^18^. We emphasise that this method can be combined with other SEM functions, such as chemical composition^19^, crystal orientation^20^, and lattice strain^21^ analyses in bulk samples. The method allows in-depth study of microstructures including spin textures, and is widely applicable to problems at the forefront of physics, chemistry, and materials science, for which understanding the interplay between magnetism and crystallographic microstructure is essential.

## Results

### Observation of magnetic domains using bulk samples

First, we describe SEM observations acquired from a bulk Fe-40 at% Pt specimen, which exhibits high magnetocrystalline anisotropy^18^. Figure 2a shows an SEM image acquired with an ILD. The surface plane is normal to the easy magnetization axis, that is, the [001] direction in the L1_0_-type ordered structure. Mazy magnetic domains are clearly observed all over the regions. This result is consistent with Lorentz and Kerr microscopy studies for crystals with high magnetocrystalline anisotropy^9^. In addition to the maze pattern, in this bulk form, the observation reveals a spotty contrast (indicated by single arrows in Fig. 2a) that represents small, branching magnetic domains that are magnetised in the opposite direction (*i.e.,* a type of 180° domain). As demonstrated in other systems that exhibit uniaxial, high magnetic anisotropy, these small magnetic domains are essential for reducing the demagnetization energy^9^. The results therefore indicate that this method is able to visualise complex, hierarchical magnetic microstructures that are produced on the surface of bulk specimens.

We demonstrate the utility of the magnetic imaging with SEM and an ILD by comparing the results with other observations. Figure 2b and c show the surface topography and magnetic domain structure, respectively, imaged by conventional MFM using the same specimen as that shown in Fig. 2a. The small dots in Fig. 2b are the surface roughness caused by polishing with an Ar ion beam. In Fig. 2c the image contrast is related to the stray magnetic field out of the sample^5^,^6^. Although the small, branching magnetic domains can be clearly identified, the presence of the maze pattern is not clear. The observation nevertheless reveals an outline of the complex magnetic domain structure shown schematically in Fig. 2g. Figure 2d and e show SEM images acquired with a non-annular ETD. The field of view is the same as those in Fig. 2b and c. Because the collector bias is negative (−15 V)^17^, backscattered electrons are dominant in Fig. 2d. Note that the collector bias is an electric bias applied to the ETD, and is different from the bias applied to the specimen, as discussed in the next section. The image clearly shows the surface reliefs as small dots, which was also observed in Fig. 2b. The contribution of the secondary electrons becomes substantial when a positive collection bias is applied (Fig. 2e, collector bias 250 V). In this case, due to the deflection of secondary electrons by the Lorentz force from the stray magnetic field, the SEM image reveals information about the magnetic domains, namely type-I contrast^9^,^16^,^17^. However, as shown in Fig. 2e, the magnetic domain configuration is blurry because of the incomplete acquisition of the magnetic deflection using an ETD; that is, the observation does not allow in-depth study of the complex magnetic domain structure such as that shown in Fig. 2g. The reason for the incomplete imaging of the magnetic domains will be addressed in a later section. Figure 2f shows an SEM image obtained by using the annular ILD. With the aid of the ILD, which is positively biassed at 8 kV and efficiently collects deflected secondary electrons as discussed below, the visibility of complex magnetic domains is improved considerably compared with the results in Fig. 2e. Figure 2f shows the small, branching magnetic domains observed by MFM, and the locations of the mazy magnetic domains can be determined straightforwardly. We note that the contrast is still obscured in several portions of the boundaries in the mazy magnetic domains in Fig. 2f. However, it appears that the magnetic contrast can be further improved by optimising image acquisition conditions such as the spacing between the sample and ILD (this spacing is also known as the working distance^17^) and the kinetic energy of the incident electrons (see Supplementary Note 1).

### Kinetic energy of signal electrons that contribute to the magnetic contrast

To understand the mechanism that creates the magnetic contrast in the SEM image taken by using the ILD, we examined the energies of the signal electrons (*i.e.,* secondary and/or backscattered electrons). We can distinguish secondary electrons from backscattered electrons, both of which have escaped from the specimen, by their kinetic energy^17^,^19^. According to this classification, signal electrons with a kinetic energy of less than 50 eV are secondary electrons, whereas backscattered electrons have an energy of more than 50 eV. Thus, an electric bias (0 to 50 V) was applied to the specimen (Fig. 3f) to alter the collection efficiency of secondary electrons. As explained in the later part of this section, the applied electric bias changes the electric potential of scattered electrons within the crystal. As shown in Fig. 3a–c, the contrast of the magnetic domains (observed in a Fe-40 at% Pt bulk sample) becomes gradually more obscure as the electric bias is increased to 30 V. When the electric bias is increased further (40 and 50 V; Fig. 3d and e), the contrast of the magnetic domains disappears. The energy range over which the magnetic contrast can be observed (0–30 V) is consistent with the energies of secondary electrons (<50 eV). We therefore conclude that the magnetic contrast is mainly due to secondary electrons and it is likely that the contribution of backscattered electrons to the magnetic contrast in Fig. 3 is small.

Phenomenologically, the effect of positive electric potential can be explained based on the escape probability, the concept of which is widely accepted in conventional SEM studies^17^. This parameter represents the probability of secondary electrons that have sufficient kinetic energies escaping from the specimen. For secondary electrons, the escape probability, *p(z*), from depth position *z* (measured from the sample surface) is expressed as^17^


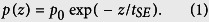


Here, *p*_0_ represents the escape probability at position *z* = 0, which depends on the work function. *t*_SE_ is a material-dependent constant representing the maximum depth that contributes to secondary electron emissions, which is only on the order of several nanometers^17^,^19^. Application of a positive electric bias to the specimen increases the effective work function, decreasing *p*_0_ in equation (1), and the escape probability, *p(z*), is suppressed accordingly. This relationship explains the observations in Fig. 3 well. Note that the incident electrons and backscattered electrons are only negligibly affected by this range of electric bias since the acceleration voltage of incident electrons (5 kV) is greater than the electric bias by two orders of magnitude.

## Discussion

As mentioned in the last section, the magnetic contrast is mainly due to the deflection of secondary electrons that have escaped from the sample. For the mazy magnetic domains, such as those shown in Fig. 2a, the in-plane component (*x-y* component) of the stray magnetic field is maximised above the magnetic domain walls. This is confirmed by simulations of the stray magnetic field for a model specimen showing the mazy pattern in Supplementary Note 2. The complex stray magnetic field makes the angular distribution of secondary electron emissions asymmetric with reference to the optical axis and position-dependent in the *x-y* surface plane. For example, in a 300-nm-thick Co-Pt film shown in Supplementary Note 2, the in-plane component of the magnetic field is significant over a range of 10 μm from the sample surface, and thus the magnetic deflection is significant in this range. In contrast, because the secondary electrons are emitted from only a limited region close to the surface (*e.g*., <2 nm)^17^, the magnetic deflection of secondary electrons due to the inner magnetization is negligible (see Supplementary Note 2). Detection of the secondary electrons (deflected by the stray magnetic field) thus produces an SEM image that reveals the magnetic domain structure.

**Figure 1:**
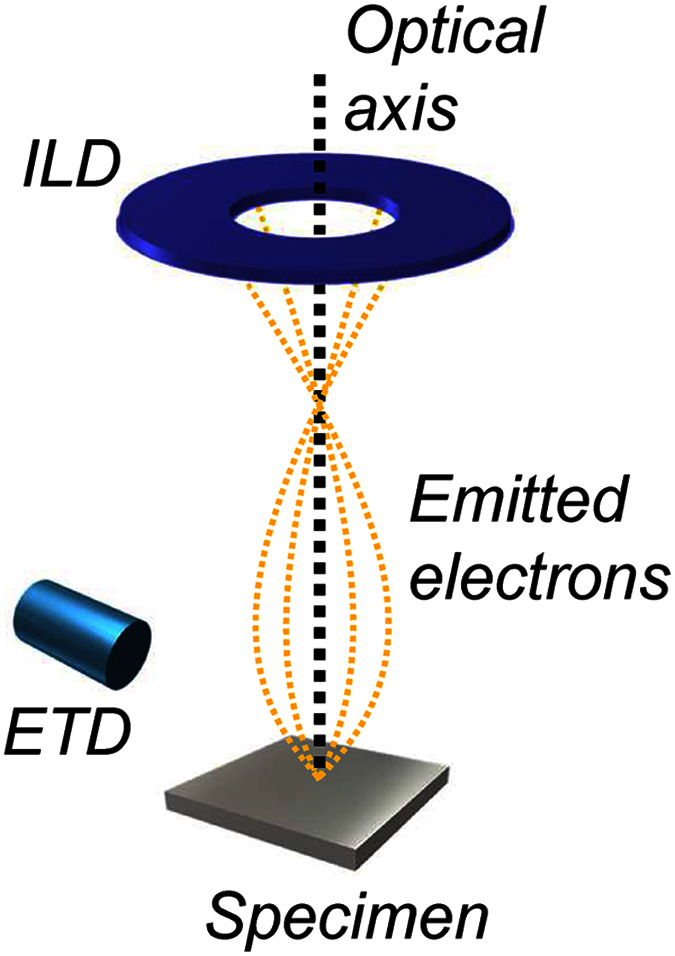
Experimental setup for magnetic imaging in SEM. The non-annular ETD is located outside the SEM column and is tilted away from the optical axis. The annular ILD is placed inside the SEM column and centred on the optical axis.

**Figure 2:**
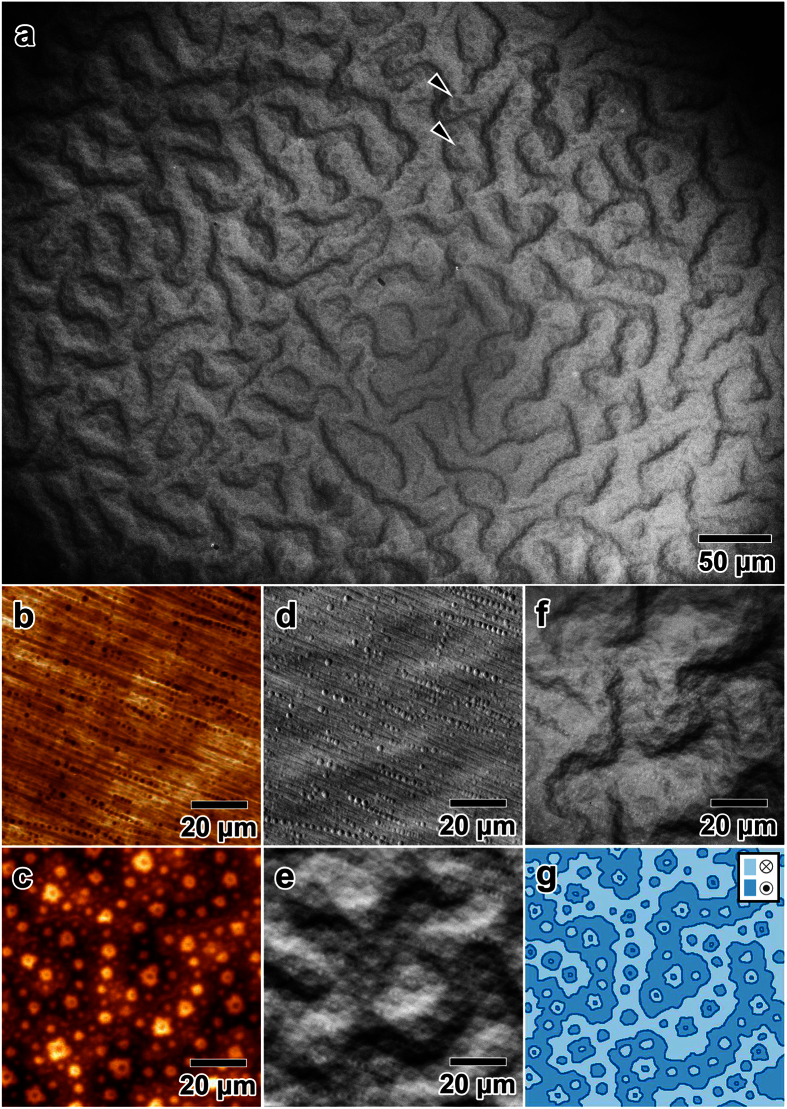
SEM and MFM images of the Fe-40 at% Pt bulk specimen. (**a**) ILD image of bulk Fe-40 at% Pt. (**b**) Surface topography and (**c**) MFM images. A macroscopic mazy pattern and spotty, closed reverse domains can be observed in the latter. (**d**) Surface topography image obtained by ETD with a collector bias of −15 V. (**e**) ETD image obtained with a collector bias of 250 V. (**f**) ILD image clearly showing the domain walls and spotty reverse domains. (**g**) Schematic illustration of the surface domain structure. Two different regions are magnetised in opposite directions as indicated by the inset legends. SEM was operated at an acceleration voltage of 2 kV and a working distance of 3.5 mm (refer to Supplementary Note 1 for the definition of the working distance).

As demonstrated in previous studies^16^,^22^,^23^, magnetic contrast can be obtained even by using a non-annular ETD (conventional type-I contrast, such as that shown in Fig. 2e), although only limited portions of the magnetic domains are visible to the ETD that is tilted away from the optical axis. In contrast, using an annular ILD makes the magnetic domains entirely visible because of the efficient collection of secondary electrons and the symmetric, on-axis geometry of the ILD (Fig. 1). In fact, the magnetic domain pattern observed using an ILD remains unchanged regardless of sample rotation (0–270°) with respect to the axis of electron incidence (Fig. 4a–d). When the ETD is used, as shown in Fig. 4e–h, the observations reveal only limited portions of the magnetic domains, and the contrast clearly depends on the detector positions that are indicated by the red arrows. The small difference in Fig. 4a–d may be due to a slight tilt in the surface and imperfections in the annular detector^24^,^25^ that could cause rotation-dependent contrast.

The effective resolution of magnetic domain observations in SEM depends on several factors (see, for example, ref. 17 for spatial resolution in SEM and ref. 22 for the resolution of type-I imaging): (1) the information volume^17^ from which secondary electrons are emitted; (2) the collection efficiency of secondary electrons with the detector; and (3) the complexity in the stray magnetic field outside the sample. Factors (1) and (2) are the microscope-dependent, and (1) and (3) are specimen-dependent. Although we need further observations to determine these factors for the specimens/microscope we used, the actual resolution in the SEM observations can be deduced from our observations. The MFM image in Fig. 2c reveals small, branching magnetic domains, the size of which is on the order of 100 nm or smaller. The small magnetic domains are also seen in Fig. 2a and f. These results indicate that the resolution of the ILD-based method can reach 100 nm or less, which is comparable to the resolution reported for conventional type-I imaging^22^. Because we used a stray magnetic field in the magnetic imaging, the contrast may be blurred for extremely small magnetic domains. Simulations of magnetic imaging will help to solve this problem.

**Figure 3:**
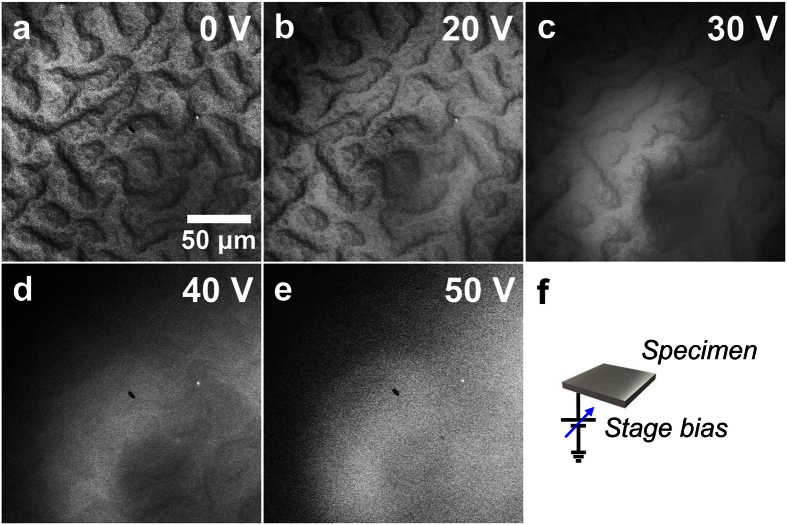
ILD images of bulk Fe-40 at% Pt specimen under elevated electric stage bias. (**a**–**e**) ILD images obtained with an elevated electric stage bias from 0 to 50 V. The magnetic contrast is weakened at 30 V and almost disappears at 50 V. (**f**) Schematic illustration of the experimental setup. SEM was operated at an acceleration voltage of 5 kV and a working distance of 7.0 mm.

**Figure 4:**
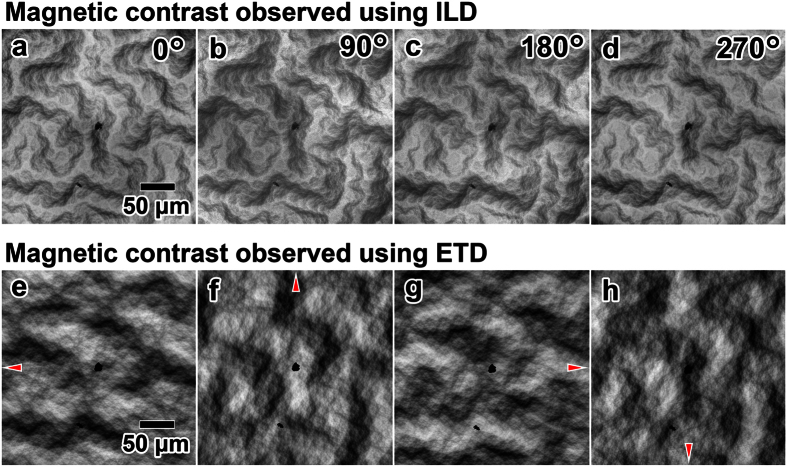
Rotation experiments for ETD and ILD images in the bulk Co-50 at% Pt specimen. (**a**–**d**) ILD and (**e**–**h**) ETD images of the sample rotated with respect to the optical axis by 90° at a time (0–270°). The ETD images show a drastic change in magnetic contrast, whereas the ILD images retain a similar contrast. All SEM images are, for simplicity, aligned so that the sample direction is unchanged, although in the experiment, the sample is rotated and the detector positon is fixed. SEM was performed at an acceleration voltage of 5 kV for (**a**–**d**) and 2 kV for (**e**–**h**), and a working distance of 10.0 mm for (**a**–**d**) and 5.0 mm for (**e**–**h**).

**Figure 5:**
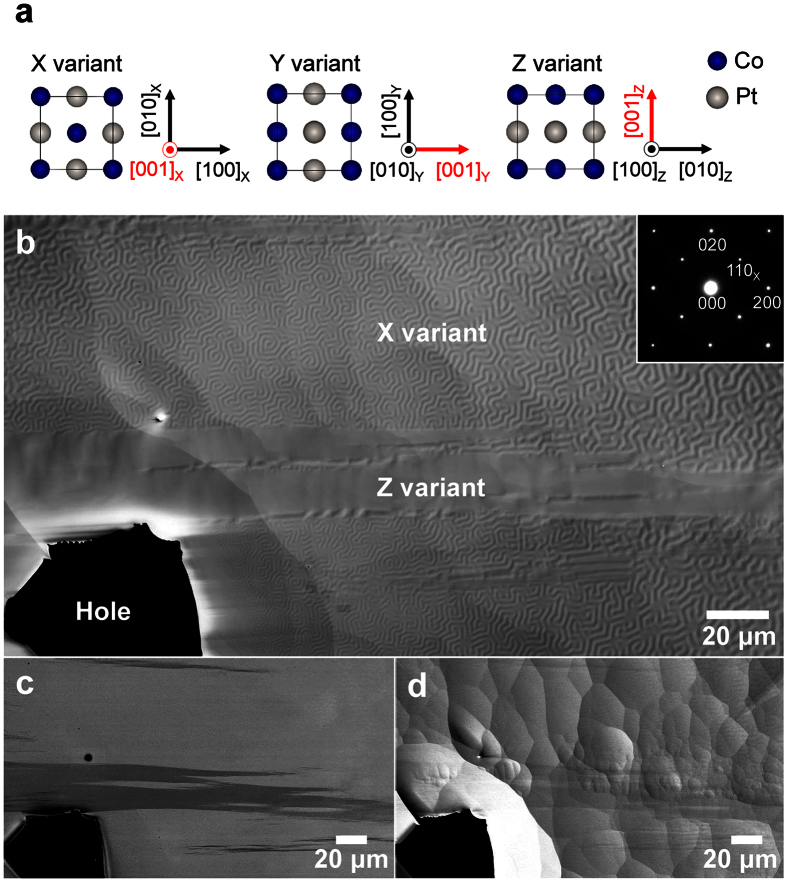
SEM images of a Co-50 at% Pt thin plate. (**a**) Crystallographic relation between the three tetragonal orientation variants in the L1_0_-type-ordered Co-Pt alloy. (**b**) ILD image including X and Z orientation variants. A mazy domain structure is clearly visualised in the X variant region. The TEM selected area electron diffraction pattern in the inset, which was obtained from the edge region that has the mazy pattern, exhibits only superlattice reflections of the X variant. (**d**) Crystal orientation image obtained by using another annular detector for backscattered electrons. Two regions corresponding to the X and Z variants have different contrasts. (**e**) ETD image showing the surface relief caused by the Ar ion beam. SEM was performed at an acceleration voltage of 5 kV and a working distance of 7.0 mm.

A big advantage of ILD-based magnetic imaging is that it can be combined with other SEM techniques, such as chemical composition analysis, crystal orientation analysis, and strain analysis. Figure 5 shows an example of the combination with crystal orientation analysis for a Co-50 at% Pt alloy that also has a large magnetocrystalline anisotropy^18^. Owing to the tetragonal L1_0_-type ordered structure, the specimen contains three orientation variants (crystallographic domains), X, Y, and Z, which can be distinguished by the orientation of the [001] direction corresponding to the easy magnetization axis (Fig. 5a). For each variant, the projections of the crystal structure from the viewing direction are shown in Fig. 5a. Figure 5b–d show the SEM images of a thin foil of the Co-Pt alloy that is also suitable for TEM observations. Note that the field of view in Fig. 5b–d contains only X and Z variants. These images were simultaneously obtained by using different detectors: the ILD for secondary electrons (Fig. 5b), an annular detector placed below the ILD for backscattered electrons (Fig. 5c), and the ETD for secondary electrons (Fig. 5d). The observations in Fig. 5b–d clearly show the magnetic domains, crystallographic orientations, and surface topography, respectively. The result shown in Fig. 5c (providing only the crystallographic information) is consistent with the conclusion that the contribution of backscattered electrons to the magnetic imaging is small (Fig. 3). Interestingly, the crystal orientation image in Fig. 5c shows that the region of the X variant (bright area in Fig. 5c) corresponds perfectly to the area showing the mazy magnetic domains in Fig. 5b. Furthermore, due to the wide-field of view of SEM, the magnetic image (Fig. 5b) explicitly shows that the width of many magnetic domains increases as the specimen thickness increases as has been reported in previous studies^9^. Thus, this method is useful for understanding the effect of the demagnetization field^9^ in thin-foil specimens. There is no magnetic contrast in the region of the Z variant, which appears to be a single magnetic domain or a simple form consisting of huge magnetic domains, and is magnetised in the in-plane direction. The image obtained with the ETD (Fig. 5d) clearly shows the roughness of the sample surface, whereas this is not as prominent in Fig. 5b. These results clearly demonstrate that the magnetic imaging using the ILD can provide multiple types of information about the microstructure in combination with other detectors.

In this study, we have demonstrated a simple method of magnetic imaging using an annular detector referred to as an ILD, which is placed on the optical axis of an SEM. The method has several advantages over other magnetic imaging techniques because it (1) reveals the magnetic domain structure on the surface of bulk samples with a resolution of 100 nm or less, (2) can view, in principle, entire magnetic domains, regardless of the sample rotation with respect to the optical axis, and (3) can be combined with other detectors and analytical instruments for conventional SEM, enabling analysis of properties, such as surface topography, composition, and crystal orientations, which affect the magnetic domain structure. There are also practical advantages, including short acquisition time (~1 min for a single acquisition) and use of conventional vacuum conditions (~1.0 × 10^−3^ Pa) that are far easier to achieve compared with SEMPA (<1.0 × 10^−7^ Pa)^7^.

As mentioned above, SEM is a surface-sensitive method. The magnetic domain structure at the surface may differ from that in the interior of bulk crystals (*e.g.*, in soft magnetic materials), and the surface domain pattern can even be modified by surface roughness^9^. Nevertheless, the method using SEM provides essential information for several applications. One important application is for the study of permanent magnets^26^, in which characterization of both magnetic domains and crystallographic microstructure is vital for understanding the coercivity mechanism^27^,^28^. Another potential application is magnetic recording^1^,^2^, in which direct observation of the surface magnetic domains (which produce a stray magnetic field representing the bit pattern) provides useful information about magnetization reversal and other such basic mechanisms, even though we use model specimens showing relatively large domains. Although TEM-based methods require an electron-transparent specimen, SEM allows for the surface-domain observations of bulk specimens and/or device configurations fabricated on a thick substrate without any polishing to prepare thin foils. This point is also beneficial for studies on materials with magnetic domain structures that are highly sensitive to strain and/or specimen shape (*e.g.,* magnetostrictive materials)^29^,^30^. The array of magnetic vortices produced in superconductors^31^,^32^, the spacing of which is on the order of 100 nm or larger, is also an important target of the method. SEM studies are expected to be useful for determining the positions of individual vortices as they produce a significant stray magnetic field in the specimen surface. We accordingly expect that SEM observations using an ILD, which reveals a surface magnetic domain structure in two dimensions, can be used in many problems at the forefront of science and technology. The method is also expected to be useful beyond the classical type-I imaging that is sensitive to magnetization in only one direction because of the asymmetric geometry of the detector.

To understand the image contrast quantitatively, the nature of the secondary electrons emitted from the magnetic sample needs to be examined in more detail. Nevertheless, the observations indicate that the method using ILD/SEM extends the applications of SEM-based magnetic imaging to many leading-edge subjects related to spin textures for which magnetic imaging using a bulk sample is essential.

## Methods

### Sample preparation and electron microscopy observations

Ingots of Co-50 at% Pt and Fe-40 at% Pt alloys were prepared by arc melting, with single crystals grown using the floating zone method. Samples were homogenised for 168 h in an evacuated quartz tube (2.0 × 10^−4^ Pa) at 1273 and 1423 K, respectively, followed by quenching in ice-water. The Co-50 at% Pt used for SEM observation in particular was treated based on two-step heat-treatment including magnetic annealing in order to obtain a homogeneous L1_0_-ordered state, the details of which have been reported^33^. After determining the crystallographic orientation by the Laue method, samples were fabricated that have a {100}_A1_ surface. Thin Co-50 at% Pt foil was prepared by mechanical polishing, dimpling, and Ar ion milling. SEM specimens were prepared by mechanical polishing and gentle Ar ion milling. The TEM selected area electron diffraction pattern was obtained by using a JEOL JEM-2000EX microscope operating at 200 kV. SEM and MFM observations were carried out using FEI Scios and JEOL JSPM-5200 systems, respectively.

## Additional Information

**How to cite this article**: Akamine, H. *et al*. Imaging of surface spin textures on bulk crystals by scanning electron microscopy. *Sci. Rep.* **6**, 37265; doi: 10.1038/srep37265 (2016).

**Publisher’s note:** Springer Nature remains neutral with regard to jurisdictional claims in published maps and institutional affiliations.

## Supplementary Material

Supplementary Information
