# Supplementary material for: Imaging of surface spin textures on bulk crystals by scanning electron microscopy
Source: Sci Rep. 2016 Nov 22;6:37265. doi: 10.1038/srep37265 (PMC5118727; doi:10.1038/srep37265)
Supplement: Supplementary Information [file srep37265-s1.pdf]

Imaging of surface spin textures on bulk crystals by scanning electron microscopy

Hiroshi Akamine[1], So Okumura[1], Sahar Farjami[2], Yasukazu Murakami[3,4], Minoru Nishida[2]

[1]*Department of Applied Science for Electronics and Materials, Interdisciplinary Graduate School of Engineering Sciences, Kyushu University, Kasuga 816-8580, Japan*

[2]*Department of Engineering Sciences for Electronics and Materials, Faculty of Engineering Sciences, Kyushu University, Kasuga 816-8580, Japan*

[3]*Department of Applied Quantum Physics and Nuclear Engineering, Faculty of Engineering, Kyushu University, Fukuoka 819-0395, Japan*

[4]*The Ultramicroscopy Research Center, Kyushu University, Fukuoka 819-0395, Japan*

Supplementary Figures

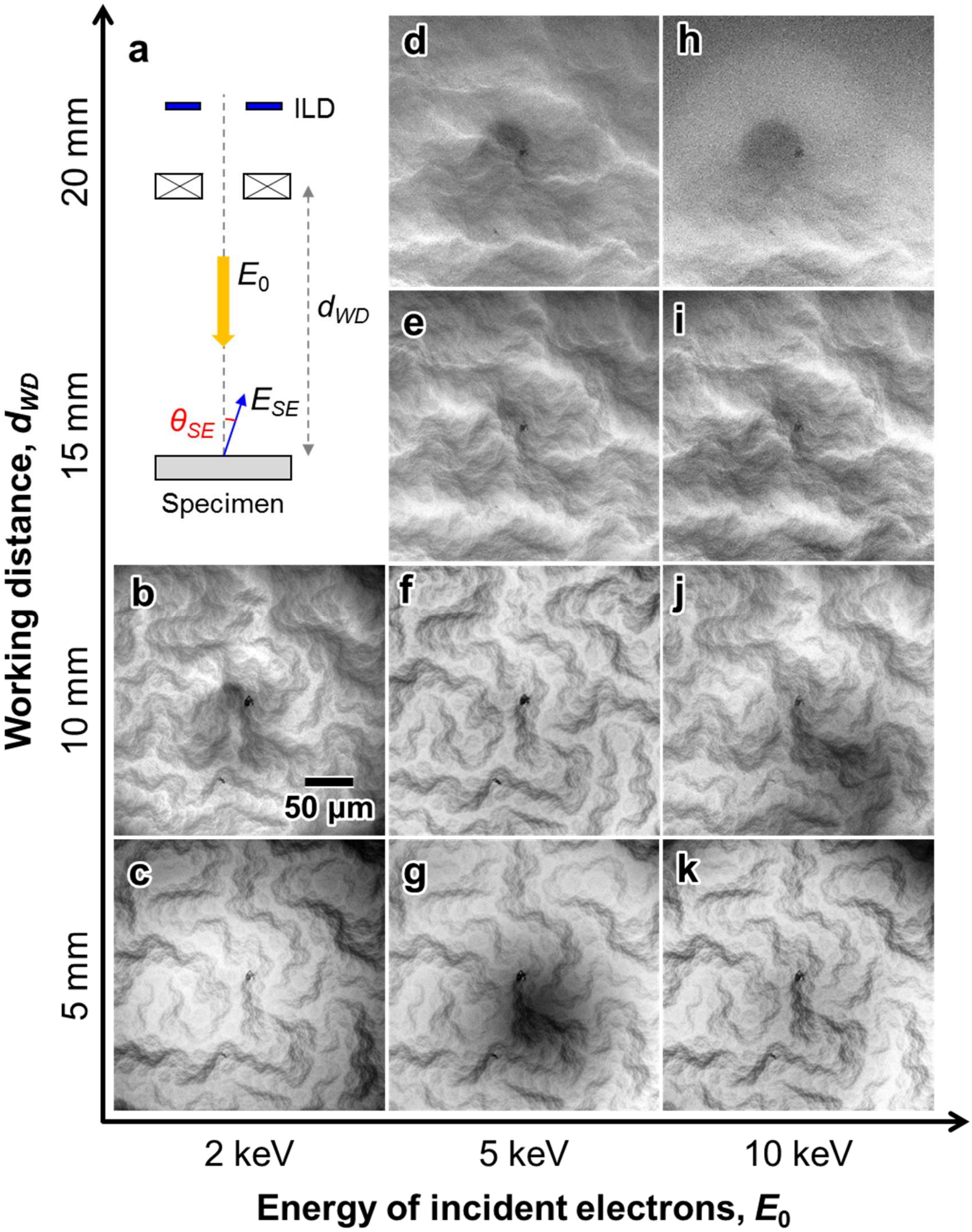

Supplementary Figure S1: Variation of the magnetic contrast with the kinetic energy of incident electrons and the working distance. **a:** Schematic of the experimental setup, where E_0 is the energy of incident electrons, E_{SE} is the energy of emitted secondary electrons, θ_{SE} is the take-off angle, and d_{WD} is the working distance, which is the distance between the pole piece and the surface of the specimen. **b-k:** ILD images obtained using different parameters.

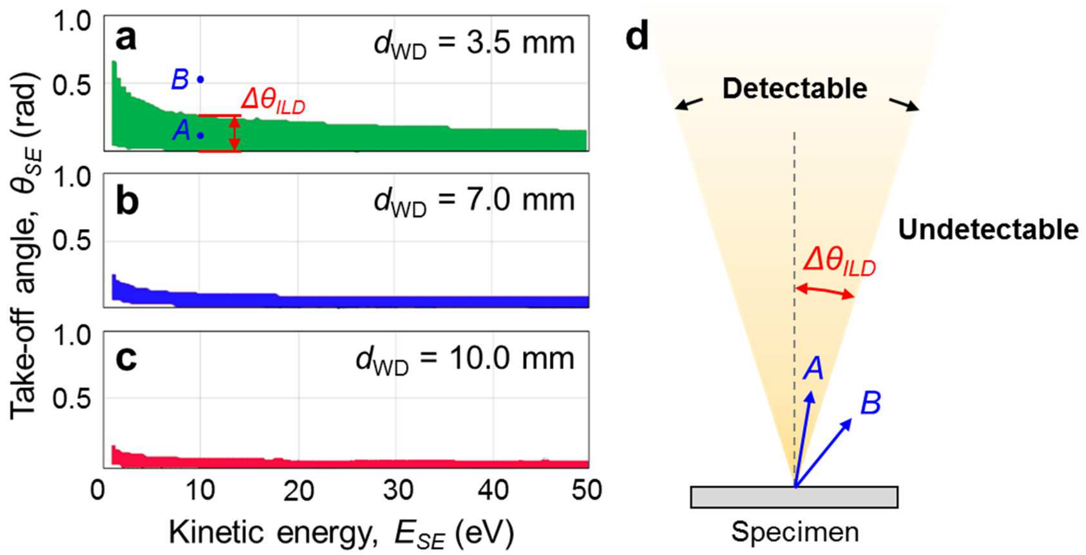

Supplementary Figure S2: Detectable range of the kinetic energy, E_{SE}, and take-off angle, θ_{SE}, of secondary electrons. **a-c:** Detectable ranges are indicated by the colored regions for each working distance. **d:** Schematic illustration of detectable/undetectable ranges of θ_{SE}. Blue arrows labeled A and B correspond to the blue points in **a**. $\Delta\theta_{SE}$ is the detectable range of θ_{SE} at E_{SE} = 10 eV.

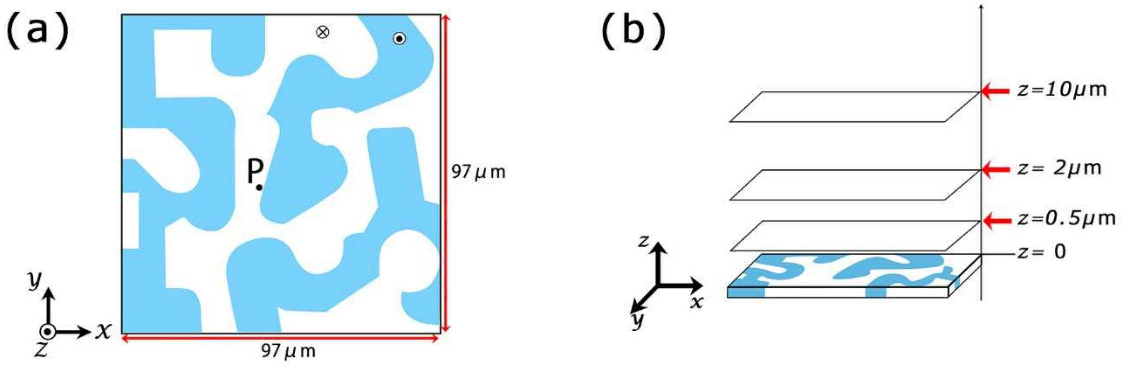

Supplementary Figure S3: Model specimen used in calculations of the magnetic field. **a**: Structure of mazy magnetic domains. **b**: Positions of *x*-*y* planes (outside the specimen) for which the contour maps of magnetic flux density were calculated.

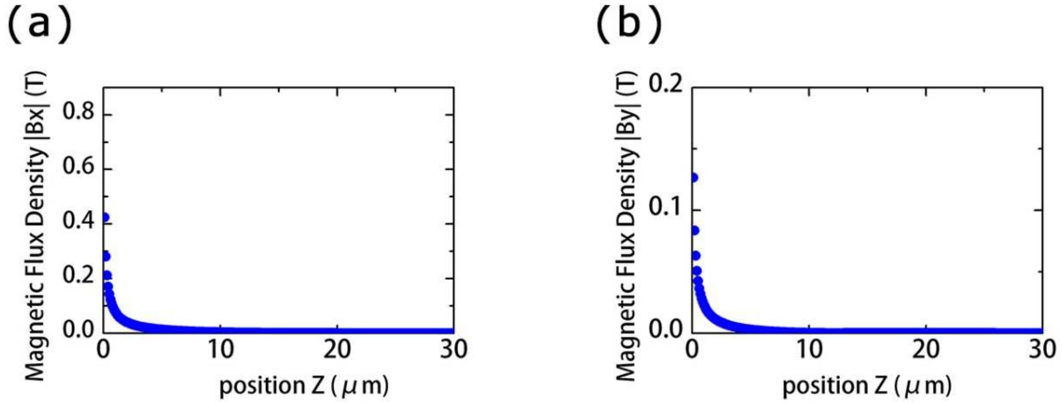

Supplementary Figure S4: Magnetic flux density (in absolute values) as a function of *z* position, which is the distance from the top surface of the model specimen. Refer to the *x*-*y*-*z* coordinate system in Supplementary Fig. S1. **a**: *x* component of magnetic flux density, B_x. **b**: *y* component of magnetic flux density, B_y.

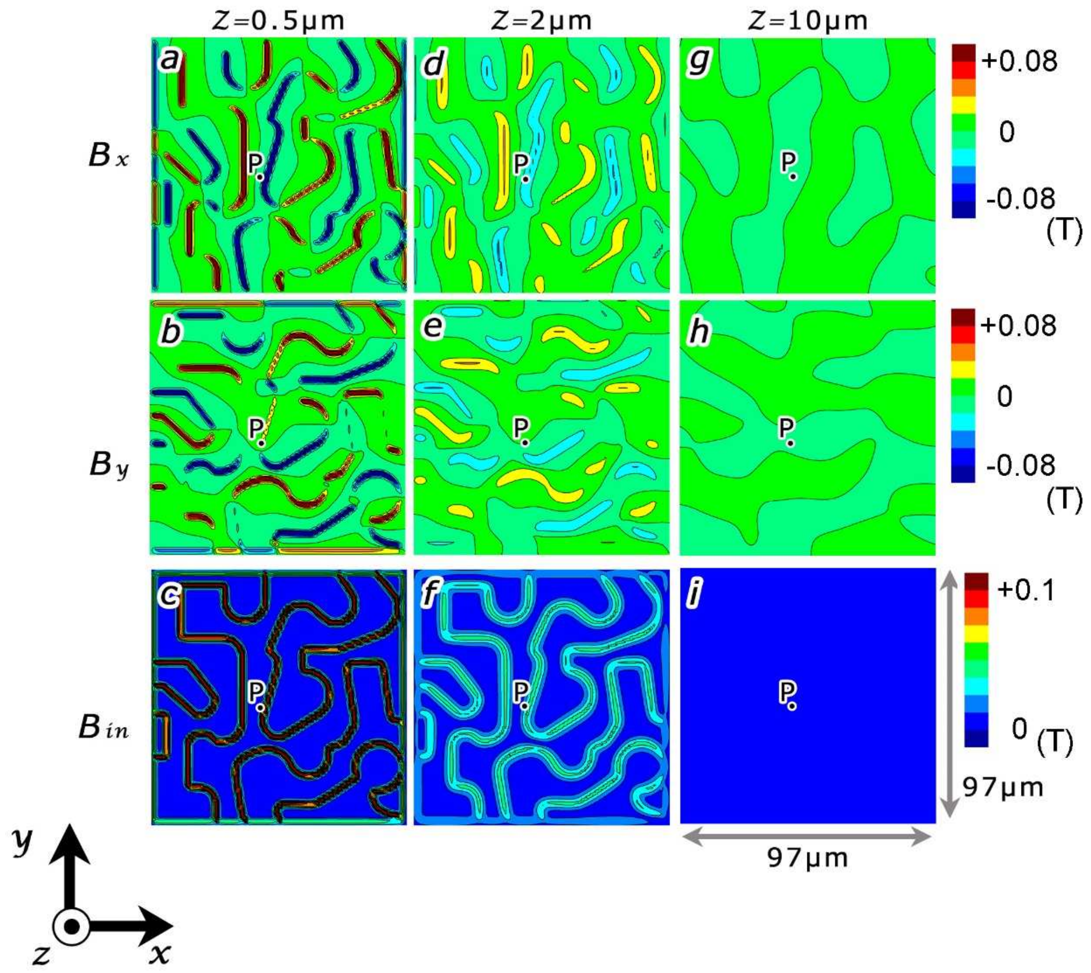

Supplementary Figure S5: Contour maps of in-plane magnetic flux density B_x, B_y, and B_{in}. See text for the definition of B_{in}. **a-c**: Calculations for the *x*-*y* plane at *z* = 0.5 μm. **d-f**: Calculations for the *x*-*y* plane at *z* = 2 μm. **g-i**: Calculations for the *x*-*y* plane at *z* = 10 μm. Refer to color bars for the magnitude of magnetic flux density, shown in teslas (T).

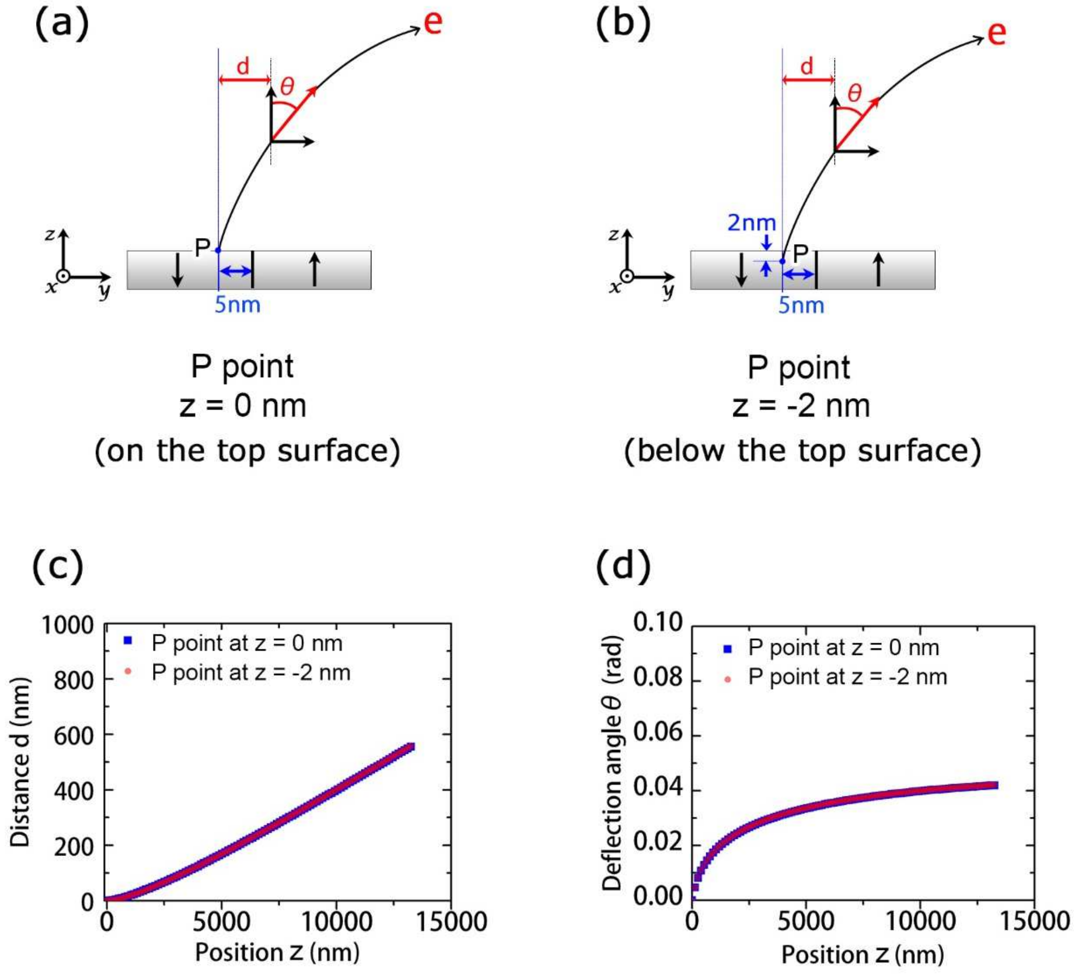

Supplementary Figure S6: Deflection of secondary electrons by the stray magnetic field. **a**: Schematic illustration showing the motion of a secondary electron generated at point P on the top surface of the model specimen (z = 0 nm). **b**: Schematic illustration showing the motion of a secondary electron generated at point P, below the top surface by 2 nm (z = -2 nm). **c**: Distance from the vertical axis crossing point P, plotted as a function of z. **d**: Deflection angle, plotted as a function of z.

Supplementary Notes

Supplementary Note 1: Effects of microscope-dependent factors on magnetic contrast in in-lens annular detector image

In this section (Note 1), we discuss several factors that may affect the magnetic contrast revealed by the annular in-lens detector (ILD) equipped with a scanning electron microscope. Two conventional microscope-dependent factors are examined: the kinetic energy of incident electrons, E_0, and the distance between the ILD and the specimen, which is also called the working distance, d_{WD}. The working distance is defined as the distance between the pole piece and the specimen surface (Supplementary Fig. S1a) [S1]. Both E_0 and d_{WD} can be directly controlled. E_0 is determined by the acceleration voltage and d_{WD} can be varied by moving the specimen along the electron incidence direction.

Other factors that need to be examined are the motion of emitted secondary electrons, which can be expressed by kinetic energy, E_{SE} (<50 eV [S1]), and take-off angle, θ_{SE} (angular deviation from the surface normal) (Supplementary Fig. S1a). A constant θ_{SE} indicates the initial angle of a secondary electron that is emitted from the specimen, with reference to the electron incidence direction. θ_{SE} is different from the deflection angle, θ, which represents deflection by the Lorentz force due to the stray magnetic field of the specimen (Supplementary Fig. S6), and is discussed in Supplementary Note 2 in detail. Because E_{SE} and θ_{SE} are determined by complicated excitation and random walking processes within the specimen, the values of E_{SE} and θ_{SE} are normally given by probability distributions. For example, the most probable value of E_{SE} is 1 to 5 eV for metals, and that of θ_{SE} is 0 rad (normal to the surface) [S1]. Supplementary Refs. [S1, S2] discuss typical forms of the distributions that are a good approximation for a variety of alloys and compounds, including the specimen discussed below. Next, in Note 1, we discuss the relationship between the image contrast and the parameters E_0, E_{SE}, θ_{SE}, and d_{WD}.

Supplementary Figs. S1b–S1k show the variation in the magnetic contrast of mazy magnetic domains with changes in the kinetic energy of incident electrons, E_0 (horizontal axis), and the working distance, d_{WD} (vertical axis). The specimen is a bulk $L1_0$-type CoPt that has uniaxial, high magnetocrystalline anisotropy [S3]. The observations reveal that the magnetic contrast is more sensitive to d_{WD} than to E_0 under these experimental conditions. The brightness is inhomogeneous in some of these images (dark region in Supplementary Fig. S1g), although understanding the complex imaging mechanism remains challenging. The magnetic contrast weakens significantly at d_{WD} of 15 mm, and almost vanishes at 20 mm. The weak dependence on E_0 can be explained by the independence of the probability distributions of E_{SE} and θ_{SE} of the energy of incident electrons, E_0 [S2]. In contrast, the strong dependence on d_{WD} is mainly due to the change in the detection range of θ_{SE}.

To provide further information about the magnetic imaging using the ILD and scanning

electron microscopy using the FEI Scios system in this study, Supplementary Figs. S2a–S2c show the detection ranges of θ_{SE} and E_{SE} with the ILD, calculated for several values of d_{WD} supplied by FEI. For each image, the colored region represents the secondary electrons collected by using the ILD. Supplementary Figs. S2a–S2c show only whether a secondary electron emitted with a specific E_{SE} and θ_{SE} is detected on the ILD, and thus include no information about how many secondary electrons with each (E_{SE}, θ_{SE}) condition are emitted. This information is given by the probability distributions. For example, at d_{WD} = 3.5 mm, a secondary electron emitted with E_{SE} = 10 eV and θ_{SE} = 0.2 rad (denoted as A in Supplementary Fig. S2a) is within the colored region, and thus can be detected (schematic illustration in Supplementary Fig. S2d). Another secondary electron with E_{SE} = 10 eV and θ_{SE} = 0.2 rad (denoted as B) is outside of the detectable range of θ_{SE} (represented by $\Delta\theta_{ILD}$), and thus cannot be detected on the ILD. The detectable range of θ_{SE} at any E_{SE} is reduced by an increase of d_{WD}. In contrast, the detectable range of E_{SE} (0–50 eV), over which a secondary electron can be collected by using the ILD, remains unchanged for the variation in d_{WD}. This result indicates that the change in the magnetic contrast shown in Supplementary Fig. S1 (d_{WD}-dependence of the magnetic contrast) is mainly due to the contributions from the detectable range of θ_{SE}.

Further considerations, such as the effects of the specimen tilt, the magnetic lens field, and the inner and outer angle of the ILD on the observations, are still needed for a complete understanding of the magnetic contrast shown in Supplementary Fig. S1. Nevertheless, based on these results and discussion, the magnetic contrast is (1) weakly dependent on the kinetic energy of incident electrons, E_0, and (2) highly sensitive to the working distance, d_{WD} (the distance between the ILD and the specimen). The conclusion is that (2) arises from the strong d_{WD}-dependence of the detectable range of the take-off angle, θ_{SE}, for the calculations in Supplementary Fig. S2a–S2c.

The series of observations in Supplementary Fig. S1 is also helpful for optimizing the observation conditions. The observations in Figs. S4a–S4d in the main text were performed under the same conditions as those in Supplementary Fig. S1f, which shows a good image contrast compared with those for the other conditions.

Supplementary Note 2: Calculation of magnetic field of the mazy magnetic domains

To understand the magnetic deflection of secondary electrons, which is responsible for the magnetic domain observations using an annular in-lens detector (ILD), the magnetic field of the mazy domains was calculated in three dimensions. We used a model specimen that provided mazy magnetic domains (Supplementary Fig. S3a). The model specimen was 97 × 97 μm in the

x-y plane, and 300 nm in the z axis, using the x-y-z coordinate system in Supplementary Fig. S3a. The model specimen was made of two types of magnetic domains, shown as light blue and white regions, which were magnetized in the +z and -z directions, respectively. The saturation magnetic flux density of the specimen was assumed to be 1.01 T, which corresponds to the value for Co-50 at% Pt alloys [S3]. In the following calculations, the width of the magnetic domain walls was assumed to be zero because we were mainly interested in the motion of secondary electrons that were deflected by a stray magnetic field. The magnetic field was calculated by using commercial computer code ELF/MAGIC (ELF Co.), which uses the integral element method.

For secondary electrons moving in the z-axis, x and y components of magnetic flux (B_x and B_y) are responsible for the magnetic deflection exerted by the Lorentz force. Accordingly, we calculated the absolute values of B_x and B_y as a function of the position in the line parallel to the z-axis crossing the point P (Supplementary Figure S3a). The point P was 5 nm away from the magnetic domain wall, as in Supplementary Figs. S6a and S6b. Near the top surface of the model specimen, the absolute value of B_x exceeds 0.4 T (Supplementary Fig. S4a). Even the absolute value of B_y is larger than 0.1 T (Supplementary Fig. S4b). As shown in the below, this extent of stray magnetic field can deflect secondary electrons significantly. However, both B_x and B_y are reduced markedly as the observation point moves away from the top surface. For example, B_x shows only a negligible value on the order of 0.001 T at observation point z = 10 μm. The results indicate that, when the effect of a lens magnetic field can be ignored, the secondary electrons can be severely deflected only in a limited region near the surface, for example, at z < 10 μm for this model specimen.

As discussed in the main text, the deflection of secondary electrons by a stray magnetic field can be a primary source for the magnetic contrast in SEM observations. This is attributed to type-I imaging, which explains the magnetic contrast in conventional SEM images, acquired by using a classical asymmetric detector [S1]. The mechanism can also be valid for observations using a symmetric detector ILD. To demonstrate the effectiveness of type-I imaging using an ILD, we compare the original magnetic domain structure (Supplementary Fig. S3a) and the contour maps of the x-y components of magnetic flux density. The contour maps are calculated for three x-y planes located in z positions of 0.5, 2, and 10 μm (Supplementary Fig. S3b). Note that z = 0 represents the top surface of the model specimen. As shown in Supplementary Fig. S3, the three parameters of the in-plane magnetic flux density, B_x, B_y, and $B_{in} = \sqrt{B_x^2 + B_y^2}$ were plotted in these x-y planes.

First, we focus on the results for z = 0.5 μm. As shown in Supplementary Figs. S5a and S5b, both B_x and B_y are maximized as positive values at the locations of several magnetic domain walls (red regions). Interestingly, parameters B_x and B_y can be minimized (maximized as negative

values) in the other positions of the magnetic domain walls (blue regions). The results indicate that secondary electrons are deflected to the largest extent at those locations of magnetic domain walls. In other words, the magnetic contrast in SEM images reveals the positions of magnetic domain walls.

For additional information, the contour map of B_{in} (Supplementary Figs. S5c) shows an almost perfect correspondence between the in-plane magnetic flux density (maximized/minimized regions) and the magnetic domain walls shown in Supplementary Fig. S3a. The results demonstrate the usefulness of the type-I imaging for revealing the positions of magnetic domain walls. For the z = 2 μm plane, the magnitude of the in-plane magnetic flux density is reduced significantly, although the maximized/minimized regions can still be seen in the locations of magnetic domain walls (Supplementary Figs. S5d–S5f). The in-plane flux components become almost negligible at z = 10 μm (Supplementary Figs. S5g–S5i).

Assuming that a SEM image is acquired by using a classical non-symmetric detector located in the y-axis, type-I imaging should be highly sensitive to B_x. Thus, the SEM image reveals magnetic domain walls that are indicated in red and blue in Supplementary Fig. S5a, whereas those in Supplementary Fig. S5b may be obscured. In contrast, when a symmetric ILD is used, the observations are sensitive to both B_x and B_y, and this can be an advantage in using an ILD for type-I magnetic imaging.

To examine magnetic deflection that may occur in the specimen, Supplementary Fig. S6 compares the motions of secondary electrons that are deflected by the x-component of the magnetic flux (B_x). In the interior region of the specimen, B_x is related to the x-component of spontaneous magnetization (M_x) and the x-component of the demagnetization field (H_{dx}). However, the model specimen in Supplementary Fig. S1a, which was assumed to be magnetized only in the z-axis (either +z or –z), does not show the x-component, M_x. Nevertheless, the secondary electrons may be deflected by the demagnetization field, H_{dx}. We assumed that secondary electrons were generated at the P points indicated in Supplementary Figs. S3a and S3b, and emitted in the z-axis with a kinetic energy 5 eV, which is a typical value for secondary electrons in SEM [S1, S2].

To discuss the extent of the magnetic deflection, we use the parameters (1) distance from the vertical axis through point P, d, and (2) deflection angle, θ: refer to Supplementary Figs. S6a and S6b. The blue dots in Supplementary Figs. S6c and S6d represent the calculations assuming point P on the top surface, where z = 0 nm. For comparison, the red dots in Supplementary Figs. S6c and S6d show the calculations assuming point P inside the model specimen, where z = -2 nm. The blue dots almost coincide with the red dots in the calculations of d and θ: See Supplementary Figs. S6c and S6d, respectively. These results indicate that the contribution of magnetic deflection in the specimen is negligible in this model specimen, and thus the deflection is mainly due to the

stray magnetic field outside. Therefore, we conclude that the magnetic contrast) in the SEM images (*i.e.,* mazy magnetic domains shown in the main text) is mainly due to the stray magnetic field that exists outside the specimen.

Supplementary References

S1. Reimer, L. *Scanning Electron Microscopy, 2nd Edition* (Springer, New York, 1985).

S2. Goldstein, J., *et al*. *Scanning Electron Microscopy and X-Ray Microanalysis, 3rd Ed.* (Springer, New York, 2003).

S3. Vlasova, N. I., Kandaurova, G. S. & Shchegoleva, N. N. Effect of the polytwinned microstructure parameters on magnetic domain structure and hysteresis properties of the CoPt-type alloys, *J. Magn. Magn. Mater.* **222,** 138-158 (2000).

S4. Hubert, A. & Schäfer, R. *Magnetic Domains, 2nd Edition* (Springer, New York, 1998).

Acknowledgements

The authors are grateful to FEI Company for the calculation in Supplementary Figure S2 and helpful comments and to Ms. A. Sato for the calculation in Supplementary Figures S4–S6.
